# Differential immunological responses in lamb rumen and colon to alfalfa hay and wheat straw in a concentrate-rich diet: insights into microbe-host interactions

**DOI:** 10.1128/msystems.00483-24

**Published:** 2024-09-17

**Authors:** Kefyalew Gebeyew, Hui Mi, Yong Liu, Yongbin Liu, Biao Wang, Teka Feyera, Tan Zhiliang, Zhixiong He

**Affiliations:** 1 CAS Key Laboratory for Agro-Ecological Processes in Subtropical Region, National Engineering Laboratory for Pollution Control and Waste Utilization in Livestock and Poultry Production, Hunan Provincial Key Laboratory of Animal Nutritional Physiology and Metabolic Process, Institute of Subtropical Agriculture, Chinese Academy of Sciences, Changsha, Hunan, China; 2 University of Chinese Academy of Sciences, Beijing, China; 3 School of Life Sciences, Inner Mongolia University, Hohhot, Inner Mongolia, China; 4 Inner Mongolia Academy of Agricultural and Animal Husbandry Sciences, Hohhot, Inner Mongolia, China; 5 Department of Animal Science, School of Environmental and Rural Science, University of New England, Armidale, New South Wales, Australia; California State University Stanislaus, Turlock, California, USA

**Keywords:** concentrate-rich diet, interaction, lamb, microbiome, transcriptome

## Abstract

The impact of a concentrate-rich (CR) diet on the gut microbiome and epithelium homeostasis is well documented. However, it has not been systematically studied whether and how host-microbial interaction contributes to the immune homeostasis in the rumen and colon of lambs fed alfalfa hay and wheat straw, alone or combined, in a CR diet. In all, 63 lambs (initial body weight, 16.69 ± 1.50 kg) were randomly allotted to three dietary groups, each consisting of three pens with seven lambs per pen. Over 14 weeks, the lambs were fed diets as follows: 60% concentrate supplemented with either 40% wheat straw (WG), 20% alfalfa hay combined with 20% wheat straw (MG), or 40% alfalfa hay (AG). The present findings showed that lambs in the AG group had greater (*P* < 0.05) IgG and lower (*P* = 0.067) tumor necrosis factor-alpha concentrations relative to those in the MG and WG groups. The 16S rRNA analysis highlighted that various bacterial phyla and genera in the rumen and colon preferentially degrade fiber and starch derived from alfalfa hay and wheat straw. The weighted gene co-expression network analysis revealed that the bacterial genera from the *Firmicutes* are broadly associated with genes involved in various signaling pathways, underscoring the potential role of *Firmicutes* as key drivers of host-microbial interactions under the present feeding conditions. These findings shed light on the fact that the rumen and colon immune homeostasis is distinctively influenced by diets of alfalfa hay, wheat straw, or their combination in a CR diet. Further studies should examine the prolonged effects of replacing wheat straw with alfalfa hay in a concentrate-rich diet formulated to provide equivalent neutral detergent fiber levels. This could reveal how various forage fibers influence host-microbial interactions and gut health.

**IMPORTANCE:**

In contemporary feedlots, a growing trend is to feed animals a concentrate-rich (CR) diet that could disrupt the synchronized interplay between microbes and host metabolism, leading to altered metabolic functions. Wheat straw and alfalfa hay have different levels of protein and neutral detergent fiber, each with varying rates of digestion. It is unclear how including alfalfa hay and wheat straw, alone or combined in a CR diet, influences the host-microbial consortia and immune homeostasis. Herein, we showed that rumen and colon showed differential immune responses to the alfalfa hay, wheat straw, or both. Bacterial genera preferentially degrade fiber and starch derived from alfalfa hay, wheat straw, or both. Bacterial genera from *Firmicutes* phylum play a pivotal role in driving the host-microbial interactions, as indicated by their extensive association with genes across various signaling pathways.

## INTRODUCTION

The microbial community in the fore and hindgut of ruminants is reportedly shown to have multiple metabolic functions in the host, including fiber degradation and immune regulation (1, 2). These microbial communities are highly dynamic and can be significantly influenced by various feeding strategies, including feeding high levels of concentrate diets, a trend in the modern livestock industry. Diets rich in grains have been reportedly associated with microbial community imbalances, leading to reduced bacterial diversity and functionality (3, 4). Such disruptions can negatively impact gut health, as indicated by decreased pH levels, the accumulation of endotoxins, impaired fiber degradation, and changes in the short-chain fatty acid (SCFA) profiles (5). In addition, high-grain diets have been shown to severely damage the epithelial lining of the foregut and hindgut in dairy cows (2), further exacerbating microbial imbalance and their effects on host health or microbe-host interactions.

The interplay between microbial community and host metabolism is crucial for maintaining overall health and functionality, influencing a wide range of physiological, metabolic, and immunological processes. For instance, Malmuthuge et al. (6) have shown that the impact of microbes on the regulation of the rumen transcriptome is estimated to be more than 10%, highlighting the significant role of microbes in regulating host gene expression. Likewise, the gut microbiota is capable of regulating about 10% of the intestinal transcriptome in mice (7). These microbe-host interactions are dynamic, changing with developmental stages and dietary modifications. Microbial-derived SCFAs, such as butyrate and acetate, have been shown to mediate the biological processes involved in the growth of the ruminal epithelium in lambs fed a starter diet (8), highlighting the crosstalk of microbe-host in regulating several physiological processes. Moreover, dietary supplementation with conjugated linoleic acid in preweaning lambs has been shown to promote microbiota-driven transcriptional regulation in ruminal epithelial development (9). Despite these insights, the systemic study of the microbial-host interaction, particularly in response to feeding high levels of concentrate diets, remains limited. Most current researches focus on growth stages or starter diets, leaving a significant gap in understanding how these interactions influence hot metabolism under different dietary conditions.

Approximately 3 million tons of alfalfa and 4.6 million tons of wheat are produced annually in the Inner Mongolia Autonomous Region (10), making their by-products valuable feed resources for the livestock sector. Nutritionally, wheat straw is characterized by low levels of protein, along with a high amount of neutral detergent fiber (NDF) that digests slowly. On the other hand, alfalfa hay is known for its high protein content and easily digestible NDF with a rapid digestion rate, indicating significant differences in nutrient content and NDF degradation characteristics. The inclusion of wheat straw, corn stover, alfalfa hay, and bulb beet as a source of NDF in the total mixed ration of dairy cows has shown comparable results in phenotype traits when formulated to provide equivalent NDF levels (11). This suggests that the fibers from various forages can modulate the rumen environment and phenotype traits effectively. However, the specific effects of adding alfalfa hay and wheat straw to a concentrate-rich diet on phenotype traits, host-microbial consortia, and immune functions remain unclear. We assumed that the inclusion of these forages in a concentrate-rich diet may differentially affect immune homeostasis in the rumen and colon by modulating microbial-host interaction. Our study focuses on both the rumen and colon epithelia, recognizing their importance as key sites for microbe-host metabolic interactions and their diverse contributions to overall host health. By examining these tissues, we aim to enhance our understanding of how these interactions regulate immune homeostasis in both the foregut and hindgut regions in lambs-fed alfalfa hay and wheat straw, alone or combined, with a concentrate-rich (CR) diet.

## MATERIALS AND METHODS

### Animals and experimental design

The feeding trial was performed for 14 weeks, including 2 weeks of acclimatization and 12 weeks of formal periods at Hulunbuir City, Inner Mongolia Autonomous Region, China. The experiment was performed from June to September 2020. In all, 63 3-month-old fat-tailed male Hulunbuir lambs, with an initial mean body weight (BW) of 16.69 ± 1.50 kg, were used to test the hypothesis. All lambs were allotted randomly to nine pens in a randomized complete block design based on initial BW. Pens were then randomly assigned to one of the three treatment groups, with each group consisting of seven lambs per pen and three pens per treatment group. The experimental diets were prepared with a forage-to-concentrate ratio of 40:60 on a dry matter basis (DM). They consisted of similar mixtures of concentrates with wheat straw, alfalfa hay, or both (on a DM basis). The lambs were fed a diet consisting of 60% barley and corn-based concentrate, along with either 40% wheat straw (WG), 20% wheat straw combined with 20% alfalfa hay (MG), or 40% alfalfa hay (AG). Lambs received their designated diets at 0730, 1430, and 2030 hours, and were provided unrestricted access to water throughout the day. Daily records of feed offered and refusals per pen were maintained to calculate individual feed intake during the entire experimental period. The diets were prepared according to the recommendations of the Feed Standard of Meat-producing Sheep and Goats of P.R. China, NY/T 816-2004 (12).

The body weight change (BWC) was taken at 2-week intervals before morning feeding. We used a ratio method to compute the individual feed intake (IFI), as described elsewhere (13). Average daily gain (ADG) was computed by subtracting the initial BW from the final BW and dividing the results by the number of days on feed. The feed conversion ratio was computed as the feed intake divided by the BWC. We adhered to the AOAC (14) standard protocol to measure the feed dry matter (DM, Method 930.15), crude protein (CP, *N* × 6.25; Method 984.13), ash (Method 942.05), and Ca and P (Method 935.13) content of the experimental diets. The neutral detergent fiber (NDF) was determined using the method described by Van Soest et al. (15), while acid detergent fiber (ADF) was analyzed according to Van Soest and Robertson (16). The nutrient levels of the experimental diets are presented in Table S1.

### Sample collection

On day 90 before morning feeding, blood was drawn from the jugular vein of each lamb into 5 mL heparinized tubes (Changsha Yiqun Medical Equipment Co., Ltd., Hunan, China) to measure the concentrations of cytokines and immunoglobulins (Ig). After centrifugation at 3,000 × *g* for 15 min at room temperature, the aliquots were collected and frozen at −80°C until analysis. Seven lambs from each group, whose final body weights were close to the group’s mean weight, were selected and humanely slaughtered by a registered veterinarian. Following evisceration, the rumen epithelium tissue was collected from the bottom side of the central sac, and colon epithelium tissue was collected from the colon mid-segment, within half an hour. Then, the tissues were washed with sterile phosphate-buffered saline (pH 7.4), instantly frozen in liquid nitrogen, and stored at −80°C for transcriptome analysis. After filtering the rumen content (the mixed rumen digest) through four layers of cheesecloth, about 10 mL from both the rumen and colon content was aseptically collected into sterile plastic tubes, snap-frozen in liquid nitrogen, and stored at −80°C for 16S rRNA sequencing.

### Detection of plasma cytokines and immunoglobulins

The concentrations of transforming growth factor β (TGF- β), glucagon-like peptide (GLP-2), interleukin-2 (IL-2), interferon-γ (INF-γ), tumor necrosis factor-alpha (TNF-α), immunoglobulin (Ig) A, IgG, and IgM in the plasma were measured using the sheep enzyme-linked immunosorbent assay (ELISA) kits following the manufacturer’s directions (Jiangsu Meimian Industrial Co., Ltd., Yancheng, China). We used ng/mL, µg/mL, or pg/mL to present the levels of Ig and cytokines. The intra-assay and inter-assay coefficient variation (CV) of ELISA kits used for cytokines and immunoglobulins were <10% and<12%, respectively.

### Genomic DNA extraction and sequencing

The E.Z.N.A. Stool DNA Kit (D4015, Omega Bio-tek, Inc., Norcross, GA, USA) was used to isolate the microbial DNA from the rumen and colon content, following the manufacturer’s guidelines. The quantity and purity of isolated DNA were verified using a NanoDrop 2000 UV–vis Spectrophotometer (Thermo Fisher Scientific, Waltham, MA, USA) and agarose gel electrophoresis, respectively. The universal primer set 338F (5′-ACTCCTACGGGAGGCAGCAG-3′) and 806R (5′-GGACTACHVGGGTWTCTAAT-3′) along with 12 bp (base-pair) unique barcodes were used to amplify the bacterial 16S rRNA gene in the V3–V4 hypervariable region and to construct an amplicon library (17). The PCRs were performed as described in our previous study (18). A 2% agarose gel electrophoresis was used to verify the PCR products, and the AMPure XT beads (Beckman Coulter Genomics, Danvers, MA, USA) were then used to purify the PCR products. The Qubit 3.0 (Invitrogen, Waltham, MA, USA) was used to quantify the PCR products, which were pooled into one sample based on equimolar concentration to construct Illumina paired-end libraries. The quantity and size of the amplicon library were determined using the Illumina Library Quantification Kit (Kapa Biosciences, Woburn, MA, USA) and an Agilent 2100 Bioanalyzer (Agilent, Santa Clara, CA, USA). The libraries were sequenced on the NovaSeq PE250 platform (Illumina Technologies Co., Ltd, San Diego, CA, USA).

### 16S rRNA sequencing and data analysis approaches

The QIIME data analysis package (version 1.9.1) was used to perform the quality control of raw data in FASTQ files as described in our previous study (19). Clean reads were then clustered into operational taxonomic units (OTUs) at a 97% similarity cutoff using USEARCH (version 10). The representative read of each OTU was annotated and aligned to the SILVA 16s rRNA database (version 138.1) (20) using the RDP classifier algorithm with a confidence threshold of 80% (21). The population richness (Chao1) and evenness (Shannon index) were performed using the QIIME2 (available at https://library.qiime2.org/). Principal coordinates analysis (PCoA) based on the weighted UniFrac distance was used to assess beta diversity across the three treatment groups.

### RNA extraction, sequencing, and library preparation

Trizol reagent (Invitrogen, Carlsbad, CA, USA) was used to isolate total RNA from the rumen and colon epithelium tissue as per the manufacturer’s direction. The ND-1000 NanoDrop Spectrometer and Agilent 2100 bioanalyzer (Thermo Fisher Scientific, MA, USA) were used to quantify the total RNA concentration and purity, respectively. The RNA integrity was checked using agarose-formaldehyde (1%) gel electrophoresis. Samples with RNA integrity number values of greater than 7 were used for RNA-seq library construction. TruSeq RNA Library Prep Kit v2 (Illumina, CA, USA) was used to construct the library for RNA seq and enrich poly(A)-tailed host mRNA with oligo(dT) beads. The BGIseq 500 platform was used to sequence the library at BGI Tech Solutions Co., Ltd. (BGI-Shenzhen, China) and generate single-end 50-base reads as per the manufacturer’s directions.

### Quality control analysis and mapping

The Trimmomatic module in the SOAPnuke (v1.5.2) tool was employed to filter the raw reads (22). Raw reads were flagged as low quality and excluded from the data sets if they (1) contained an adapter (2); had greater than 50% of ambiguous sequences labeled as “N” (3); and had >20% of bases with a quality score <10. High-quality reads were mapped against the *Ovis aries* reference genome (Oar V3.1) using HISAT2.v2.1.0 (23) with the default parameters. The expression levels of host genes were calculated based on the number of fragments per kilobase of exon per million fragments mapped (FPKM).

### An integrating transcriptome and microbiome analysis using the WGCNA approach

We used weighted gene co-expression network analysis (WGCNA) approaches to uncover the interaction of the metagenomics and tissue transcriptome (generated from the same samples) data (24). All expressed protein-coding genes (10,146, FPKM > 1.0) in the rumen and (12,202, FPKM > 1.0) in colon tissue samples obtained from all experimental lambs were used in the WGCNA (R package v3.4.1). Briefly, the “pickSoftThreshold” function in the “WGCNA” package was used to build a gene co-expression network according to the co-expression/correlation modes among genes. We generated 19 (rumen) and 14 (colon) modules using the “blockwiseModules” function based on the following parameter setting: minimum module size of 20, the merged cutoff of 75%; and pick-soft thresholding powers of 18 and 24, respectively, and the identified clusters of densely interconnected genes (gene modules) were presented using a hierarchical clustering method. We then computed the Pearson correlation coefficients between the targeted traits and eigengenes of each gene module. The gene modules were declared as statistically associated with the targeted traits at *P* < 0.05. Thus, we found that the light-cyan and blue modules were strongly associated with the bacterial genera in the rumen and colon, respectively, and these modules were used for further correlation analysis to identify the degree of association between bacterial taxa and individual gene modules.

### Association of abundance of bacteria with the gene module

Genes from the light-cyan and blue modules—negatively and positively associated with the bacterial genera and significantly enriched in pathways—were subjected to correlation analysis. The analysis was performed using Spearman’s rank correlation coefficient, and the results were visualized with a network heatmap in the R package (Omics studio, LC-Bio Technology Co., Ltd., Hangzhou, China). Results with a *P* < 0.05 were declared as statistically significantly associated.

### The functional analysis

Database for Annotation, Visualization, and Integrated Discovery (DAVID) (v2022q3) (Huang et al., 2009) was used to perform the Kyoto Encyclopedia of Genes and Genomes (KEGG) pathways enrichment (25). The KEGG pathways were declared as significantly enriched biological functions at *P* < 0.05.

### Statistical analysis

Statistical analyses were applied using the SPSS version 23.0 (SPSS Inc., Chicago, IL, USA) and OriginPro 2023b. Each pen was used as an experimental unit for measuring body weight and feed intake. Meanwhile, individual lamb was considered an experimental unit for the remaining variables, which were measured for each animal. The one-way analysis of variance (ANOVA) with GLM procedure was employed to analyze the body weight change, individual feed intake, and plasma indices between the groups. First, the normality and homoscedasticity of data were verified using Shapiro–Wilk and Levene’s tests, respectively. Then, the differences between dietary group mean were determined using Tukey’s post hoc tests. Given that the relative abundances of bacterial phyla and genera were not normally distributed, we used the Wilcoxon rank-sum test to analyze the differential abundances between the dietary groups. The statistical differences and tendencies were regarded at *P* < 0.05 and 0.05 ≤ *P* < 0.1, respectively. The means ± standard error of the mean (SEM) was used to present the results.

The model used in the experiment was:


Yijk=µ+τi+βj+εijk

where Yijk was response variables, µ was the grand mean, τi was the ith treatment effects, βj was the jth block effect, and εijk was the error term.

## RESULTS

### Intake, performance, and immune indexes in plasma

**Fig 1:**
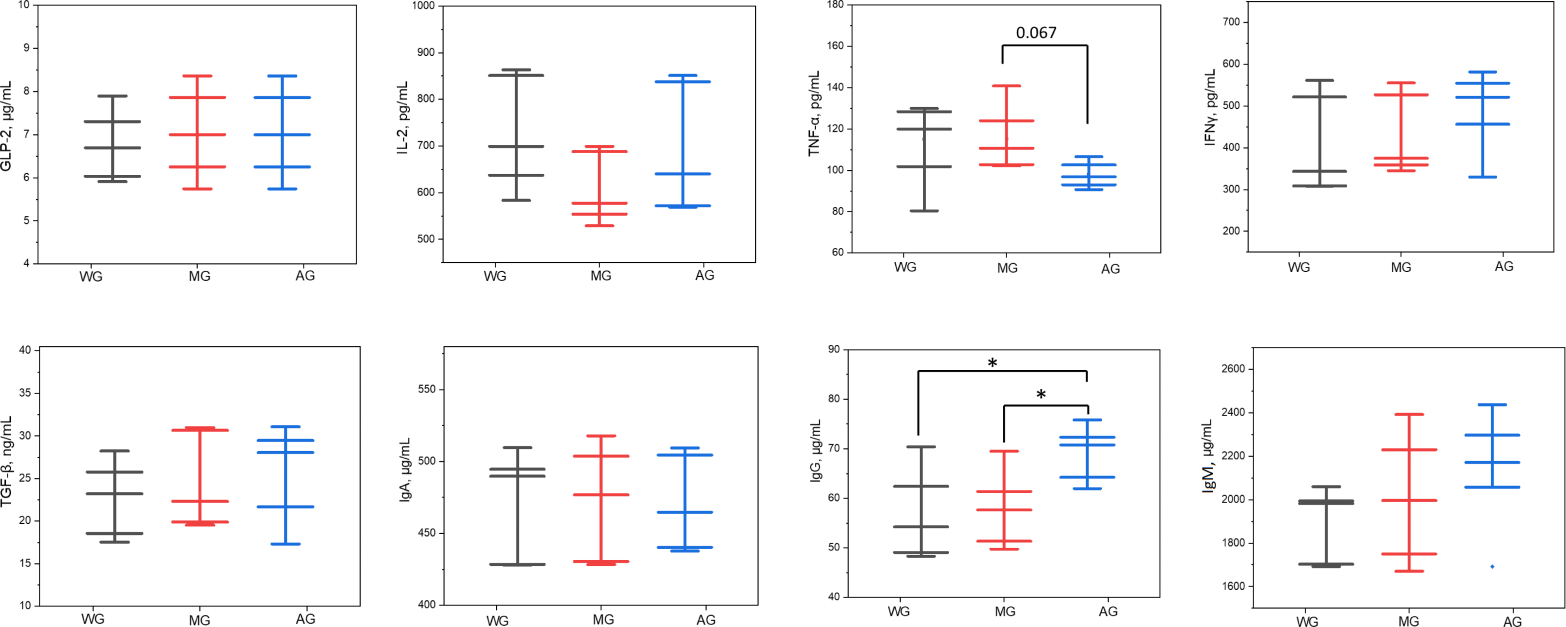
Effects of alfalfa hay, wheat straw, and their mixture supplemented with a CR diet on the concentrations of plasm cytokines and immunoglobulins in growing lambs. WG, 40% wheat straw; MG, 20% alfalfa hay and 20% wheat straw mixture; and AG, 40% alfalfa hay.

No significant differences in individual feed intake (IFI) and final body weight (FBW) were evident between the dietary treatments (Table 1). However, the body weight change (*P* = 0.092) and average daily gain (*P* = 0.093) tended to be lower in the AG group relative to the MG group (*P* > 0.05). Conversely, the feed conversion ratio tended to be greater (*P* = 0.078) in the MG group relative to the WG group. The plasma concentrations of GLP-2, IL-2, IFNγ, TGF-β, IgA, and IgM did not differ (*P* > 0.05) across the three groups (Fig. 1). The concentration of TNF-α was greater (*P* = 0.067) for the MG group than for the AG group but comparable with the WG group. Dramatic changes in the concentration of IgG were observed between the treatments, greater in the AG group (*P* < 0.05) relative to the other two groups.

**TABLE 1:** Effects of alfalfa hay, wheat straw, and their mixture supplemented with a CR diet on FBW, BWC, and IFI in growing lambs^*a*^

	WG	MG	AG	*P*-value
IBW, kg	16.60 ± 0.62	16.06 ± 1.13	17.40 ± 1.58	0.251
FBW, kg	34.77 ± 1.15	35.97 ± 1.12	34.48 ± 0.78	0.566
BWC, kg	18.17 ± 0.93	19.91 ± 1.13	17.08 ± 0.32	0.092
IFI, kg/d	1.47 ± 0.05	1.34 ± 0.06	1.37 ± 0.04	0.173
ADG, kg/d	0.22 ± 0.011	0.24 ± 0.014	0.21 ± 0.004	0.093
FCR	6.67 ± 0.435	5.55 ± 0.377	6.51 ± 0.188	0.074

^*a*^ The WG: 40% wheat straw; MG: 20% alfalfa hay and 20% wheat straw mixture; and AG: 40% alfalfa hay. FBW: final body weight; BWC: body weight changes, IFI: individual feed intakes, ADG: average daily gain; FCR: feed conversion ratio.

### Profiling of microbiota composition in the rumen and colon

**Fig 2:**
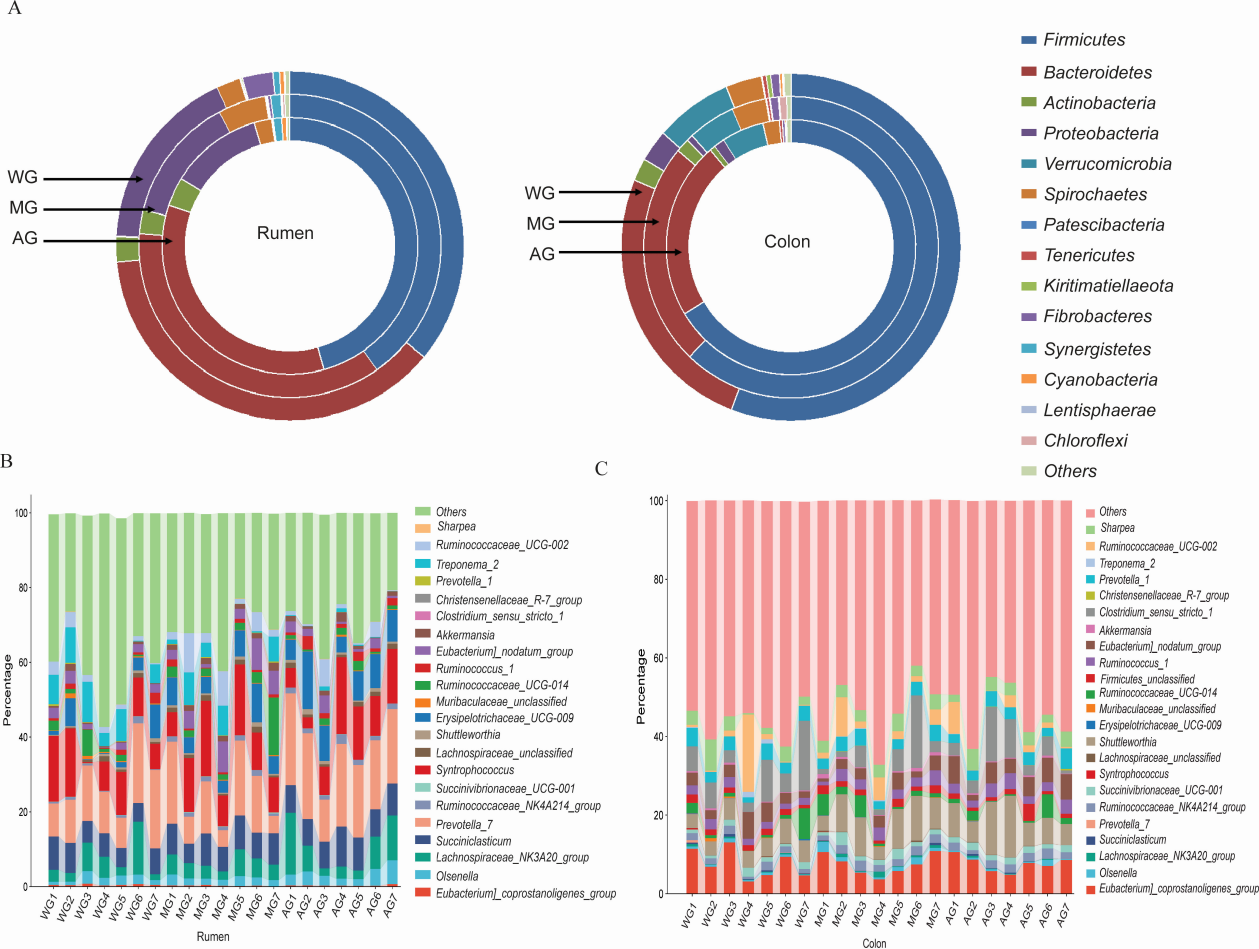
Effects of alfalfa hay, wheat straw, and their mixture supplemented with a CR diet on the relative abundance of bacterial phyla (**A**) and genera (**B, C**) in the rumen and colon of growing lambs.

The effects of wheat straw and alfalfa hay, alone or in combination, on the profiles of ruminal bacterial taxa were evaluated. The rumen and colon content microbiota results showed that the Chao1 and Shannon indices were similar across the three groups (Fig. S1). However, dramatic changes in the compositions of phyla and genera were evident in both tissues across the three dietary groups (Fig. 2A and B). Markedly, the relative abundances of *Firmicutes* and *Cyanobacteria* in the rumen for AG were greater than for the WG and MG groups, respectively (Table S2A). The MG group had a greater (*P* < 0.05) abundance of *Spirochaetes* compared with the other two groups. Compared with the WG group, the abundance of *Synergistetes* was greater in the MG group (*P* < 0.05), while the abundance of *Fibrobacteres* was lower in the AG group (*P* < 0.05). In the colon, the abundances of *Actinobacteria* and *Patescibacteria* for AG and *Proteobacteria* and *Cyanobacteria* for MG were lower (*P* < 0.05) than for the other two groups (Table S2B). Compared with that of the AG group, the MG group had a greater (*P* < 0.05) abundance of *Fibrobacteres*. In addition, while the abundance of *Verrucomicrobia* was not detected in the rumen, it was present and higher in the colon.

For ruminal genera, lambs in the WG group had greater (*P* < 0.05) relative abundances of *Eubacterium*_coprostanoligenes_group*,* Erysipelotrichaceae_UCG-009*, Prevotella_1*, and *Sharpea* compared with that in the MG group (Table S3A)*. The* abundances of *Muribaculaceae_unclassified, Eubacterium_nodatum_group, Christensenellaceae_R-7_group,* and *Treponema_2* for the MG were higher (*P* < 0.05) than for the WG or AG groups (Table S3B). The abundances of *Ruminococcaceae_NK4A214_group, Shuttleworthia,* and *Ruminococcaceae_UCG-014* were marked by the largest changes (*P* < 0.05) in the AG compared with the WG or MG groups. The abundance of *Prevotella_7* was profoundly depleted in the colon but was greater in the rumen. In the colon, lambs in the WG had a greater abundance of *Prevotella_7* (*P* < 0.05) than those in the AG group but were comparable with the MG group. Compared with that of the AG group, the MG group had greater (*P* < 0.05) abundances of *Lachnospiraceae_NK3A20_group* and *Syntrophococcus* but was comparable with the WG group. The *Lachnospiraceae_unclassified*, *Ruminococcus_1*, and *Clostridium_sensu_stricto_1* abundances for the AG group were greater (*P* < 0.05) than for the WG or MG groups.

### The interaction of the microbiome and host transcriptome in the rumen and colon using the WGCNA analysis approach

**Fig 3:**
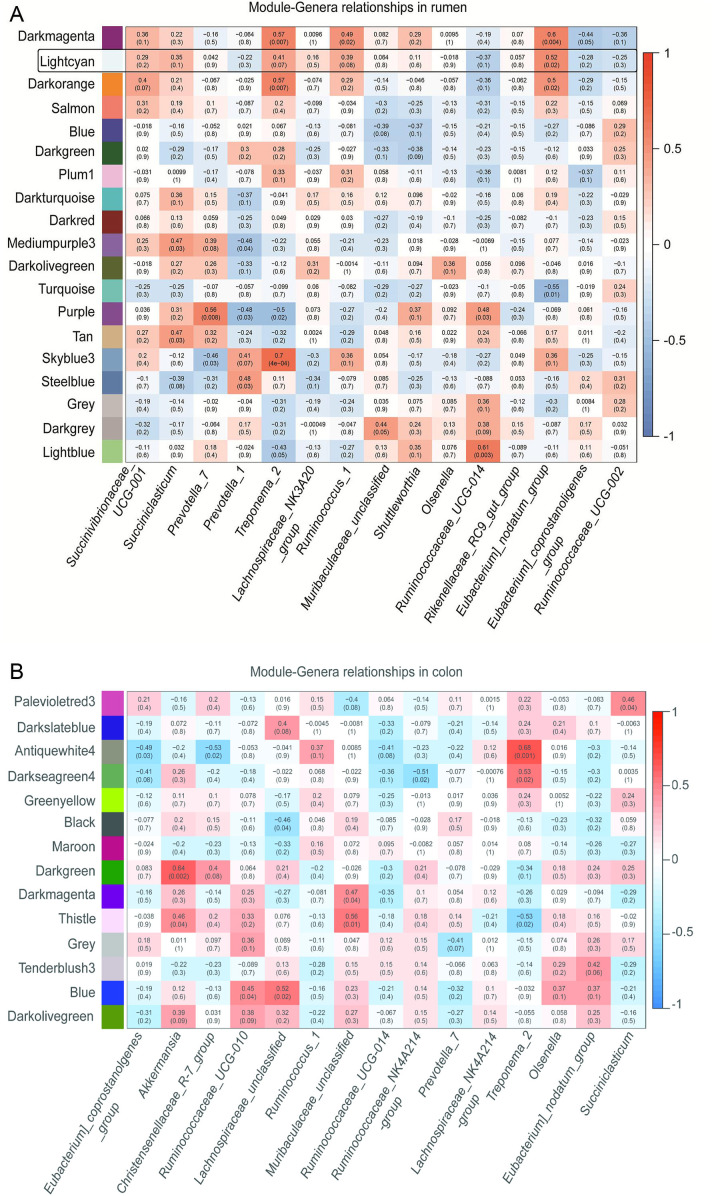
Association of host transcriptome and bacterial genera in the rumen (**A**) and colon (**B**) using the WGCNA approach. Each module name is shown on the left side, each trait is shown at the bottom, and the strength of the correlation is shown as a bar on the right in varying intensities of orange (positive correlation, *r* > 0) or blue (negative correlation, *r* < 0). The numerical values within a square represent Pearson correlation (upper value) and *P*-value (lower value).

**Fig 4:**
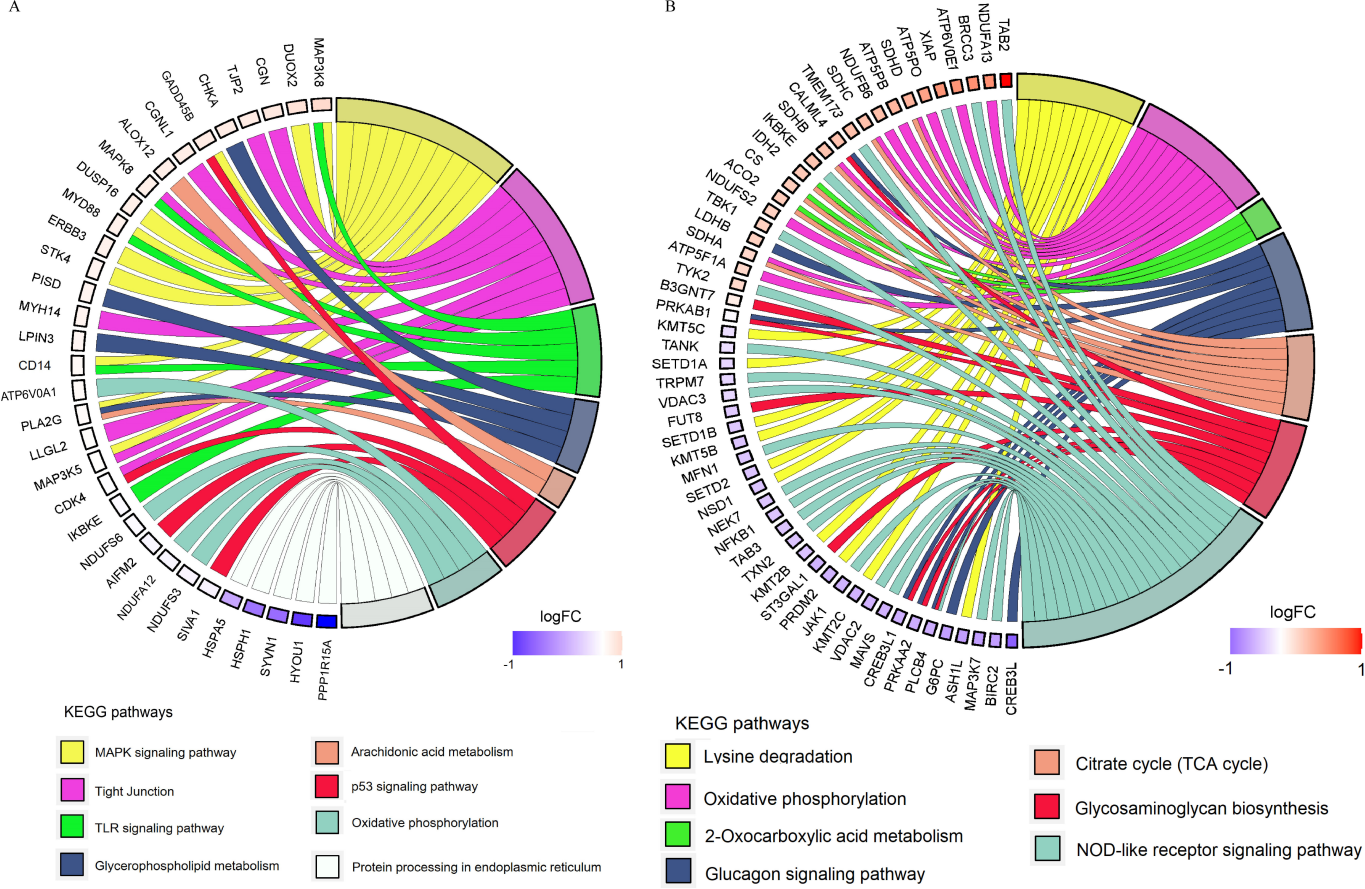
Enriched pathways in the light-cyan and blue modules from the rumen (**A**) and colon (**B**), respectively, as identified by DAVID. The pathways were considered significant at *P* < 0.05.

**Fig 5:**
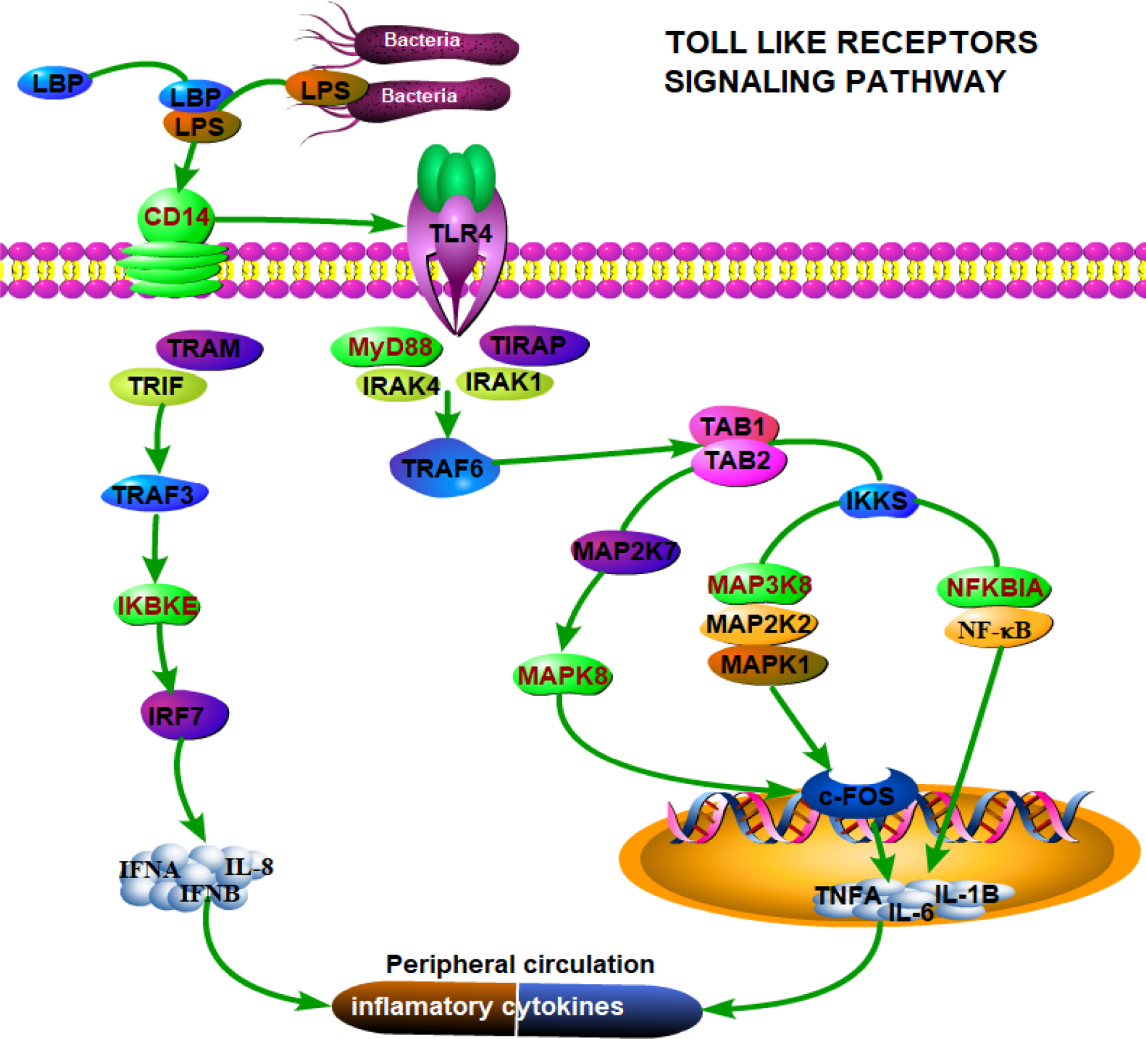
Schematic overview of toll-like receptors signaling pathway enriched in a light-cyan module from the rumen. Proteins/genes with red indicate that the gene is detected in the light-cyan module, and genes with black color are not detected in the light-cyan module.

**Fig 6:**
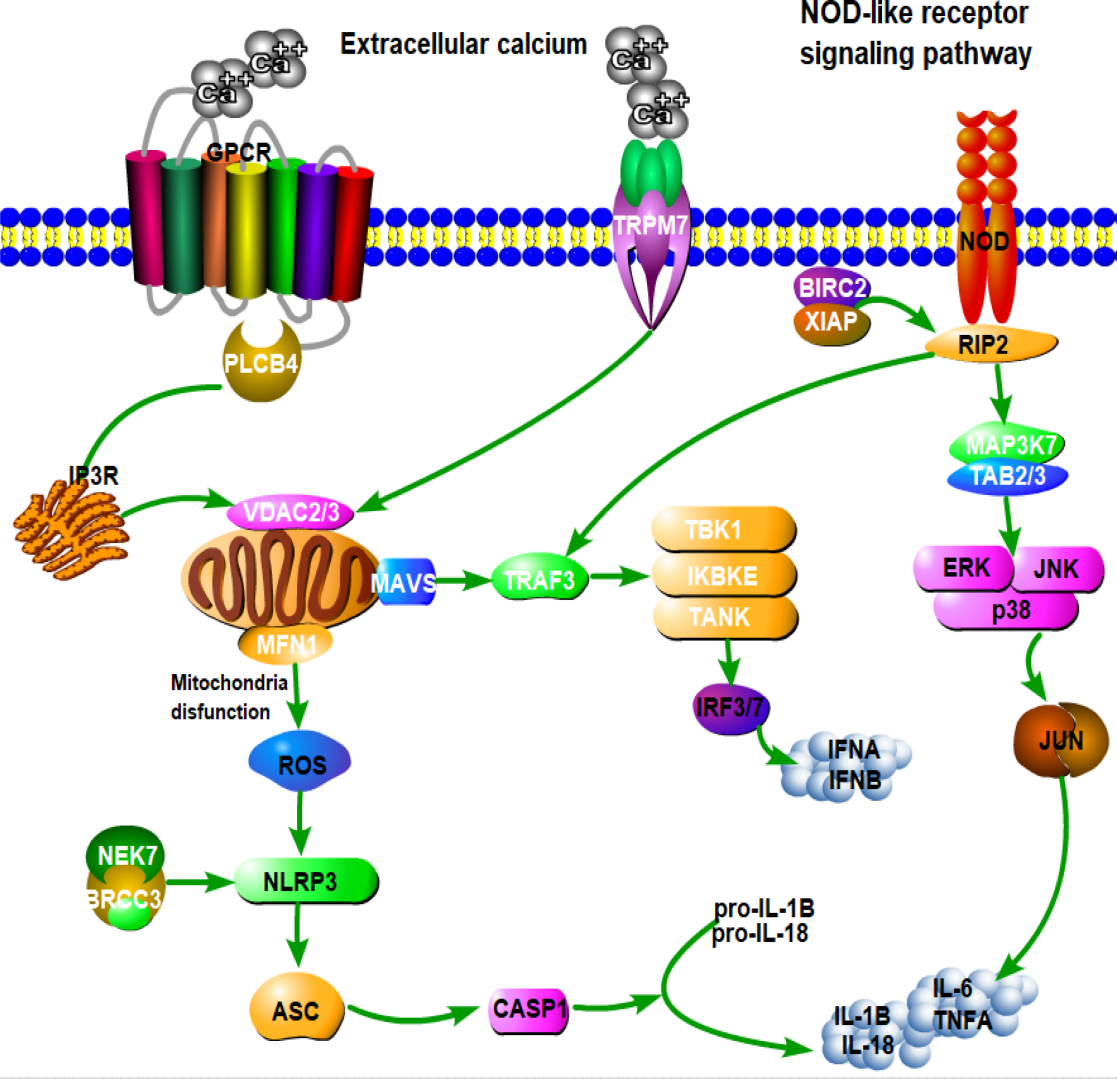
Schematic overview of NOD-like receptors signaling pathway enriched in the blue module from the colon. Proteins/genes with a white color indicate that the gene is detected in the blue module, and genes with a black color are not detected in the light-cyan module.

**Fig 7:**
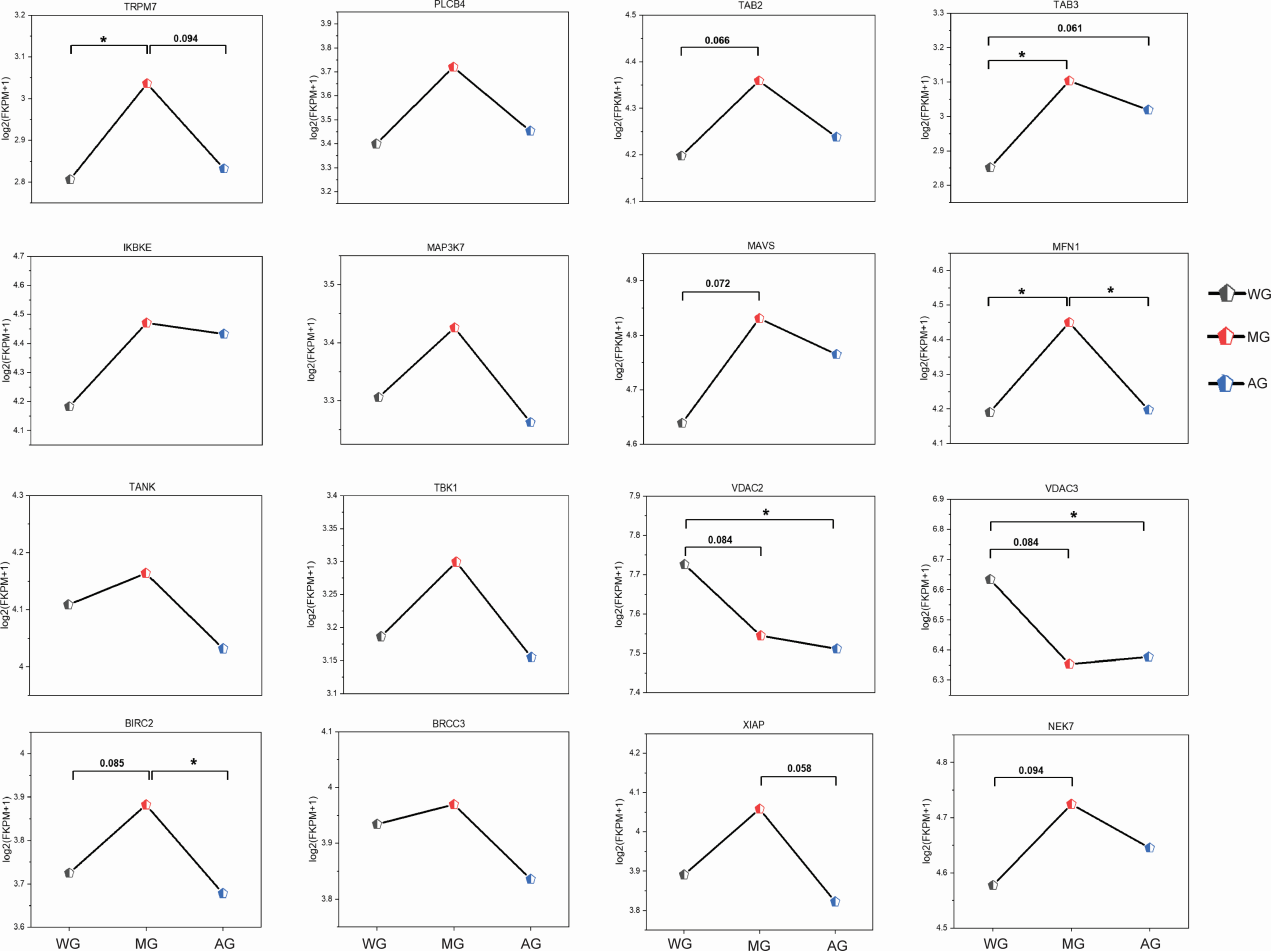
The expression [log_2_(FKPM +1)] of genes showed negative or positive associations with the individual bacterial genera in the colon. Genes involved in significantly enriched pathways in the blue module and had a significant association with the individual bacterial genera (*P* < 0.05) are displayed.

To gain better insight into the interaction between the microbiome and host transcriptome in the foregut and hindgut, we identified about 10,146 and 12,202 genes as core transcriptomes from the rumen and colon samples, respectively, using the criterion that genes must be expressed in all sample tissues and with an FPKM >1 across the three groups (Tables S4 and S5). These genes were subjected to the WGCNA approach. The results highlighted that a total of 19 and 14 modules showed various degrees of association with the abundances of bacterial genera in the rumen and colon, respectively (Fig. 3A and B; Fig. S2 and S3). The expression of genes in the light-cyan (274 genes) and blue (1,708 genes) modules showed a strong positive correlation with the bacterial genera (Tables S6 to S8). The functional analysis for the selected modules was performed using the KEGG pathway enrichment analysis. The genes in the light-cyan module from the rumen were significantly enriched in biological functions, including “The MAPK signaling pathway,” “TLR signaling pathway,” “Glycerophospholipid metabolism,” “Arachidonic acid metabolism,” and “p53 signaling pathway” (Fig. 4A). The genes in the blue module from the colon were significantly enriched with pathways related to the carbohydrate, energy, amino acid metabolism, and immune system, including “Oxidative phosphorylation,” “Glycosaminoglycan biosynthesis,” “Lysine degradation,” and “NOD-like receptor signaling pathway” (Fig. 4B). We found six genes involved in the toll-like receptor signaling pathway from the light-cyan module of the rumen: of these, the expression of MAP3K8 was greater (*P* = 0.08) in the MG group relative to the WG group (Fig. 5; Fig. S4). Likewise, we also identified 16 genes involved in the NOD-like receptor signaling pathway from the blue module of the colon (Fig. 6). Of these, eight genes in the MG group and two genes in the WG group showed significantly higher expression (0.05 ≤ *P* < 0.1), whereas the other six genes did not (*P* > 0.05) significantly change across the treatments (Fig. 7).

**Fig 8:**
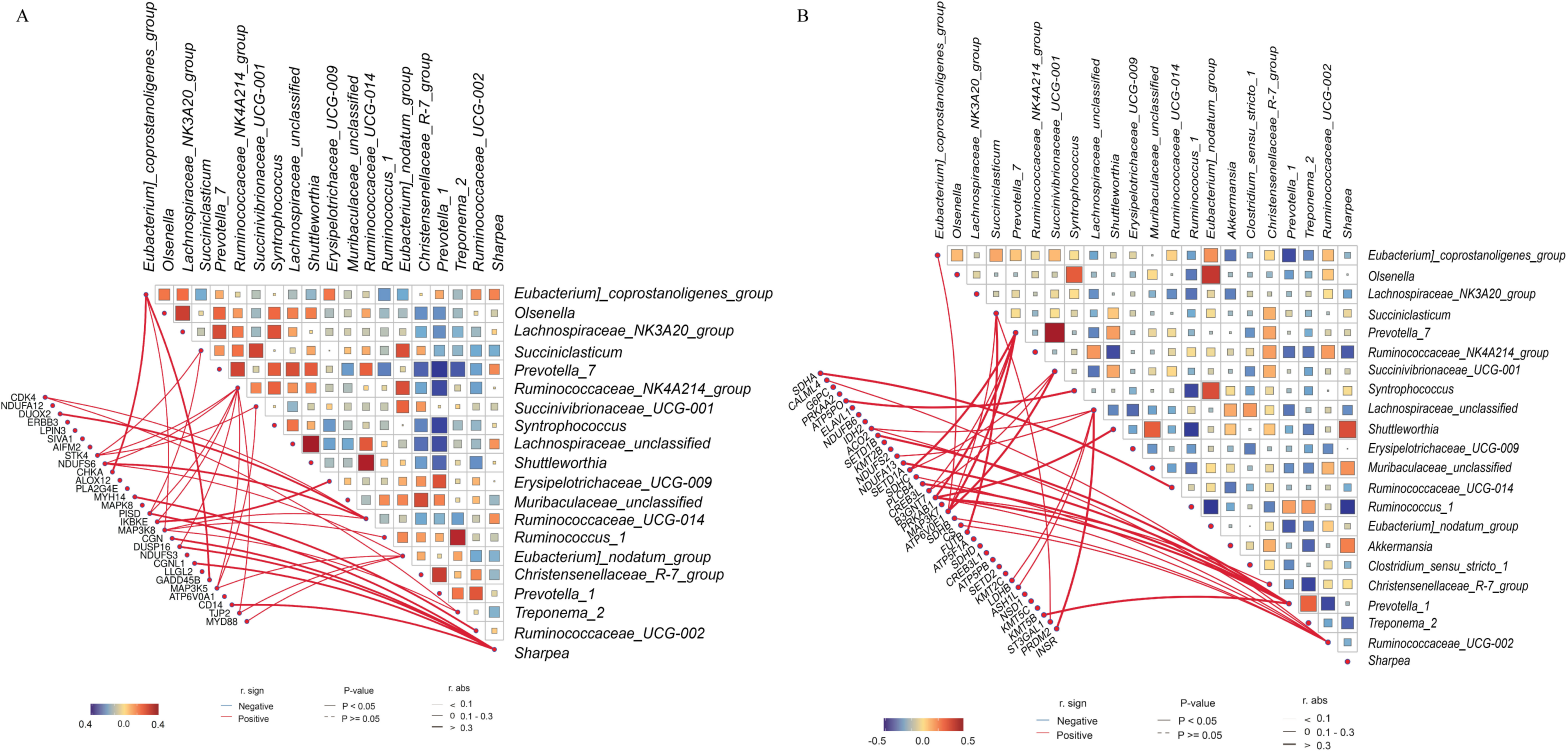
Spearman’s rank correlation between the abundances of bacterial genera and genes from the significantly enriched pathways in the selected light-cyan and blue modules of the rumen (**A**) and colon (**B**), respectively. The color within a square represents a positive correlation (orange) and a negative correlation (blue). A correlation with *P* < 0.05 is deployed in the figure.

To further identify the degree of association of each gene from the light-cyan and blue modules with the bacterial taxa, we performed Spearman’s rank correlations between the gene from the significantly enriched pathways in the selected modules and the abundance of bacterial genera. In rumen, the abundance of *Ruminococcaceae_UCG-014* showed positive correlations (*P* < 0.01) with *CDK4, DUOX2, NDUFS6*, and *MAP3K8*. The abundance of *Eubacterium_nodatum_group* had positive correlations (*P* < 0.01) with *NDUFS6, TJP2, MYD88*, and *MAP3K5* (Fig. 8A). The abundance of the *Eubacterium_coprostanoligenes_group* had a positive correlation (*P* < 0.01) with *CHKA, MAP3K8*, and *GADD45B*. The abundance of *Ruminococcaceae_NK4A214_group* showed positive correlations (*P* < 0.01) with *STK4, NDUFS6, PISD, IKBKE, DUSP16, MAP3K5*, and *TJP2*. The abundances of *Ruminococcaceae_UCG-002* and *Ruminococcus_1* had positive correlations (*P* < 0.01) with *MYH14* and *MAP3K8*, respectively. The abundance of *Sharpea* showed positive correlations (*P* < 0.05) with *PISD, CGN, DUSP16, CGNL1*, and *CD14*. The abundances of *Succiniclasticum* and *Succinivibrionaceae_UCG-001* had a positive association (*P* < 0.05) with *STK4, PISD*, or *MAP3K5*. The abundances of *Erysipelotrichaceae_UCG-009* and *Treponema_2* showed a positive correlation (*P* < 0.01) with *IKBKE* and *STK4* and *MAP3K5*, respectively.

In the colon, the abundances of *Ruminococcaceae_UCG-014, Shuttleworthia,* and *Syntrophococcus* had a positive association (*P* < 0.01) with *SDHA, PRKAB1*, and *ATP5PO*, respectively (Fig. 8B). The abundance of *Lachnospiraceae_unclassified* showed a positive correlation (*P* < 0.01) with *ACO2, NDUFA13, LDHB, ASH1L*, and *INSR*. The abundances of *Eubacterium_coprostanoligenes_group* and *Christensenellaceae_R-7_group* had a positive association (*P* < 0.01) with *ATP6V0E1* and *PRKAB1*, respectively. The abundance of *Prevotella_1* had a positive association (*P* < 0.01) with *SETD1B, SETD1A, CREB3L4, PRKAB1*, and *ST3GAL1*. The abundance of *Succiniclasticum* showed a positive correlation (*P* < 0.05) with *FUT8, ATP6V0E1,* and *PRDM2*. The abundance of *Succinivibrionaceae_UCG-001* showed a positive correlation (*P* < 0.05) with *SDHC, B3GNT7*, and *ATP6V0E1*. The abundance of *Prevotella_7* had a positive association (*P* < 0.05) with *SDHC, B3GNT7, PRKAB1*, and *ATP6V0E1*, respectively. The abundance of *Ruminococcaceae_UCG-002* showed positive association (*P* < 0.05) with *CALML4, ACO2, PLCB4, PRKAB1, SDHB,* and *CS*.

## DISCUSSION

Various forage-to-concentrate ratios have been reported to affect gastrointestinal fermentation, epithelium homeostasis, and microbiota profiles (26). Previous studies have reported that supplementing rice straw, Chinese wild rye hay, and wheat straw with the normal forage-to-concentrate ratio alters fermentation profiles and improves production (11, 27). Other researchers have evaluated the impacts of high-grain diets on ruminal microbiota and transcriptome profiles in comparison to the control diet (normal forage-to-concentrate ratio) (28, 29). However, the extent to which host-microbial interaction contributes to the phenotypic traits and immune homeostasis in the rumen and colon of lambs fed a CR diet supplemented with alfalfa hay and wheat straw, alone or in combination, has not been systematically studied. Given the negligible changes in body weight gain and feed intake at the forage-to-concentrate ratio of 30:70 in our previous study (unpublished data), we reduced the ratio to 40:60 to determine whether a higher proportion of concentrate in a total diet might limit the effects of different levels of nutrients from wheat straw and alfalfa hay on phenotypic traits. As expected, a partially replacing wheat straw with alfalfa hay in a 40:60 forage-to-concentrate (F:C) ratio showed potentially enhanced body weight change, average daily gain, and feed conversion efficiency, highlighting the influences of wheat straw and alfalfa hay mixture on the phenotypic traits become more evident at a 40:60 compared to 30:70 F:C ratio. These findings align with a previous study, which showed the inclusion of different levels of corn stover as sources of forage fiber in a 20:80 F:C ratio did not improve the average daily gain in heifers (30). Furthermore, combining rapidly digestible NDF from alfalfa and slowly digestible NDF from wheat straw in the MG diet likely promotes a more stable rumen environment and improved overall fiber digestion. This can lead to better rumen pH regulation, improved microbial protein synthesis due to the balanced supply of fermentable carbohydrates and nitrogen, and enhanced nutrient absorption and utilization, contributing to better feed efficiency and growth performance.

### Fiber- and starch-degraded bacteria show preferential enrichment in a CR diet containing alfalfa hay, wheat straw, or both

Based on the 16S rRNA analysis from the rumen and colon samples, the abundance of bacterial phyla showed preferences for breaking down fiber and fermentable substrate derived from various polysaccharides present in the forage, as evidenced by the greater abundances of ruminal *Firmicutes* and *Cyanobacteria* in AG group, *Synergistetes* and *Spirochaetes* in MG group, and *Fibrobacteres* in the WG group. These findings are consistent with previous studies, which have also noted shifts in the dominance of certain bacterial phyla and genera in response to different forage-based diets (31). Considering the role of *Synergistetes* and *Spirochaetes* in protein and amino acid fermentation and breaking down complex carbohydrates, respectively, the greater abundances of these two phyla could enhance the digestibility of the mixed diet, leading to improved feed efficiency and growth performance. The abundances of *Verrucomicrobia* and *Akkermansia,* supposed to contribute to glucose homeostasis and gut health, were greater in the colon but not detected in the rumen, indicating a region-specific role of these bacteria under the current feeding conditions. The abundances of *Eubacterium_coprostanoligenes_group* and *Prevotella_1* were enriched in the rumen but not in the colon from the WG group and are reportedly associated with reducing cholesterol levels and degrading polysaccharides, respectively (32). The enrichment of *Erysipelotrichaceae_UCG-009* in the WG of the present study is consistent with the previous study that indicated the enrichment of this bacterium in the feces of cows fed a diet containing wheat straw (33). The genera *Lachnospiraceae_NK3A20_group* and *Syntrophococcus,* both members of the *Lachnospiraceae* family, play a vital in producing butyrate and in releasing acetate through breaking down the polymeric structure of lignin (34). Although these two genera are enriched in the colon of the MG group and not in the rumen, they may not substantially contribute to the total SCFA production due to their low proportion among total bacterial populations.

We also noted that the rumen and colon bacteria showed different responses to alfalfa hay, wheat straw, and their mixtures, as exemplified by the dramatic changes in the abundance of *Shuttleworthia* in the rumen, unlike in the colon of the AG group. Given the attribution of *Shuttleworthia* in the butyrate production (35), the greater abundance of this bacterium in the AG group from the rumen can improve the synthesis of butyrate, which has a crucial role in microbial and host epithelial cell growth. The enrichments of *Ruminococcaceae_NK4A214_group* and *Ruminococcaceae_UCG-014* in the rumen, but not in the colon of the AG group, were associated with the degradation of fiber and plant cell-wall polysaccharides (29), as well as with the production of short-chain fatty acid, including butyrate (36). The abundances of *Lachnospiraceae_unclassified* and *Ruminococcus_1* have reportedly been linked to suppressing gut inflammation by modulating butyrate production (37) and other SCFAs (38). The enrichment of these two bacterial genera in the colon of the AG group is likely to alter rumen fermentation and contribute to the slight alteration of immune profiles. Overall, the enrichment of *Shuttleworthia*, *Ruminococcaceae_NK4A214_group,* and *Ruminococcaceae_UCG-014* in the rumen, along with *Lachnospiraceae_unclassified* and *Ruminococcus_1* in the colon of the AG group, correlates with a slight improvement in immune profiles in lambs fed a CR diet supplemented with alfalfa hay, as indicated by the higher IgG and lower TNF-α concentrations in plasma. In addition, the lower concentration of TNF-α in lamb from the AG group might indicate that alfalfa hay can reduce the risks of high grain-induced inflammation. In general, the variations in the microbial communities among the three dietary groups can explain the differences in FCR and BWC. The MG diet appears to support a more diverse and efficient microbial community, particularly with higher abundances of fiber-degrading and protein-fermenting bacteria. This microbial composition likely enhances nutrient digestion and absorption, leading to better feed efficiency and BWC in lambs fed the MG diet compared to those fed only wheat straw or alfalfa hay.

### The WGCNA reveals the mechanism behind microbe-host interaction in lambs fed a CR diet with alfalfa hay, wheat straw, or both

A previous study has shown that the rumen microbiota can regulate over 10% of the host’s transcriptome (6). The host-microbial crosstalk is believed to regulate various metabolic functions, including carbohydrate metabolism, amino acid metabolism, and the immune system. These metabolic pathways, which are known to respond to high-grain-based diets, are expected to be similarly responsive when lambs are fed a CR diet containing wheat straw, alfalfa hay, or both. Thus, to evaluate the host-microbial crosstalk in the rumen and colon of lambs fed a CR diet with alfalfa hay, wheat straw, or both, we applied the WCGN approach to analyze the transcriptome and metagenome data, identifying the gene modules strongly associated with bacterial genera. These identified gene modules might provide us with a better insight into how the host transcriptome and microbiome interact to regulate rumen and colon metabolism under the present feeding conditions. Therefore, we assumed that genes associated with the immune system and metabolic processes would respond to the bacterial genera significantly enriched in the rumen and colon.

Based on the WGCNA integrated analysis of rumen samples, we found that genes involved in the lipid, signal transduction, cell growth and death, and immune system were enriched in the light-cyan module, which shows both positive and negative correlation with various bacterial genera. The *Ruminococcaceae_NK4A214_group, Ruminococcaceae_UCG-014,* and *Sharpea* showed a wide range of interaction with various genes from the light-cyan module, indicating that these three bacterial genera might profoundly contribute to the host-microbial interactions in the rumen under the present feeding condition. For instance, the above-mentioned three bacterial genera showed a positive association with *IKBKE, MAP3K8,* and *CD14* genes, respectively, which are essential in the TLR signaling pathway. *MAP3K8* has been shown to regulate immune responses to pathogens and mediate TLR activation of p38α MAP kinases and ERK1/2 (39). *IKBKE* plays a crucial role in cellular immunity, the regulation of NF‐κB‐mediated inflammatory reactions, and the progression of metabolic diseases (40). *CD14* has been shown to protect against experimentally induced inflammatory bowel disease *via* sustaining intestinal integrity and barrier function (41). Considering the role of *Ruminococcaceae_NK4A214_group* and *Ruminococcaceae_UCG-014* in degrading fiber and plant cell-wall polysaccharides, these two genera are more likely to influence the *IKBKE* and *MAP3K8* through substrate derived from the degradation of starch, protein, and xylan. *Sharpea* has been implicated in producing lactate as a fermentation end-product (42) and is likely to interact with the *CD14* gene *via* lactate.

The WGCNA for the colon highlighted that genes in the blue module were related to the carbohydrate, energy, amino acid, and immune metabolism, including the NOD-like receptor signaling pathway, which is positively and negatively correlated with various bacterial genera. *Ruminococcaceae_UCG-002, Prevotella (_*7 and_1*),* and *Lachnospiraceae_unclassified* showed a wide range of interactions with various genes from the blue module, indicating these bacterial genera might be drivers of or profoundly contribute to the host-microbial interaction in the colon in response to feeding a CR diet with alfalfa hay, wheat straw, or both. Particularly, *Ruminococcaceae_UCG-002* showed a positive correlation with the genes involved in the inositol phosphate metabolism (*PLCB4*) and citrate cycle (*ACO2, SDHB,* and *CS*). *Ruminococcaceae_UCG-002* is a butyrate-producing bacterium (43), and butyrate is reported to modulate the tricarboxylic acid (TCA) cycle in the mammalian colon (44). Thus, *Ruminococcaceae_UCG-002* can influence those genes through butyrate or substrate derived from fiber degradation. *Prevotella* (*_*7 and_1) had a positive correlation with the genes involved in lysine degradation (*SETD1B* and *SETD1A*), oxidative phosphorylation (*SDHC* and *ATP6V0E1*), AMPK signaling pathway (*CREB3L4* and *PRKAB1*), and glycosaminoglycan biosynthesis (*B3GNT7* and *ST3GAL1*). Given the role of *Prevotella* in utilizing a wide range of polysaccharides and proteins (32), the substrate derived from the degradation of starch, protein, and xylan might contribute to oxidative phosphorylation, lysine degradation, and glycosaminoglycan biosynthesis *via* their respective genes. Overall, these results indicate the interaction of host transcriptome and microbes in a wide range of metabolic pathways to regulate the rumen and colon function under the current feeding condition.

### The immune homeostasis in the rumen and colon shows differential responses to alfalfa hay and wheat straw alone or combined

In the rumen, six genes associated with the TLR signaling pathway, identified from the light-cyan module, showed no significant changes among the three groups, except for MAP3K8. This highlights that the TLR signaling pathway was not profoundly affected in the rumen of lambs fed a CR diet supplemented with wheat straw, alfalfa hay, or both. On the other hand, a previous study indicated that the upregulation of *MFN2 or MFN1* expression alters mitochondrial composition and promotes mitochondrial metabolism (45).

Another study has also pointed out that feeding a high level of concentrate diet disrupts the mitochondrial function *via* Ca^2+^ overload in the mitochondria, which triggers the opening of mitochondrial permeability transition pore (mPTP)—a protein complex containing *CYPD, ANT,* and *VDAC1*—resulting in mitochondrial impairment (46). These three genes are vital for sustaining the electrochemical balance and mitochondrial homeostasis. Of the identified 16 genes associated with NOD-like receptor signaling pathways in the blue module of the colon, the expression levels of *VDAC2* and *VDAC3* were decreased in lambs from the MG group, suggesting that the mitochondria and cytosol undergo limited metabolite exchange. In addition, *MFN1, MAVS*, and six other genes associated with NOD-like receptor signaling pathways were increased in the MG group, indicating a disruption of mitochondrial homeostasis and activation of the NF-kβ signaling pathway. These results are further supported by the increased plasma concentration of TNF-α in lambs from the MG groups. Overall, the immune status of lambs fed a CR diet supplemented with wheat straw and alfalfa hay mixture might be compromised in the colon but remains unaffected in the rumen.

### Conclusion

The present study highlighted that supplementing a CR diet with alfalfa hay can slightly improve immune profiles, as evidenced by higher IgG and lower TNF-α concentrations in the plasma. A better FCR and BWC in lambs fed with a mixture of wheat straw and alfalfa hay can be associated with improved fiber utilization and a stable rumen environment, as indicated by the enrichment of fiber-degraded bacteria. The WGCNA analysis indicated that *Ruminococcaceae_NK4A214_group, Ruminococcaceae_UCG-014, Sharpea* in the rumen and *Ruminococcaceae_UCG-002, Lachnospiraceae_unclassified*, and *Prevotella* in the colon showed a wide range of interaction with genes involved in various signaling pathways, respectively. This suggests that these bacterial genera may drive or significantly contribute to the host-microbial interactions in response to feeding a CR diet supplemented with alfalfa hay, wheat straw, or both, likely by altering substrates, SCFAs, and metabolites. Rumen and colon showed differential immune responses to the alfalfa hay and wheat straw, as indicated by the unpleasant expression of several genes associated with NOD-like receptor signaling pathways in the MG group, derived from the blue module of the colon. Overall, these findings provide better insights into the roles of host-microbial crosstalk in regulating immune homeostasis in the rumen and colon of lambs under the present feeding condition. Future research should investigate the extended effects of a concentrate-rich diet where alfalfa hay substitutes wheat straw and supplies equivalent NDF, assessing its influence on host-microbial interactions. This will provide important insights into the regulatory role of different forage fibers on rumen function in lambs.

## Data Availability

The transcriptome and 16S rRNA gene sequencing data for all samples were submitted to the National Center for Biotechnology Information Sequence Read Archive (SRA) under the accession numbers PRJNA1032697 and PRJNA1032647, respectively.

## SUPPLEMENTAL MATERIAL

Supplemental Figures msystems.00483-24-s0001.pdf Figures S1 to S4.

Graphical Abstract msystems.00483-24-s0002.pdf How alfalfa hay and wheat straw alter the colon bacteria and epithelium in lambs.

Supplemental Tables msystems.00483-24-s0003.xls Tables S1 to S8.

ASM does not own the copyrights to Supplemental Material that may be linked to, or accessed through, an article. The authors have granted ASM a non-exclusive, world-wide license to publish the Supplemental Material files. Please contact the corresponding author directly for reuse.
