# Supplementary material for: Differential immunological responses in lamb rumen and colon to alfalfa hay and wheat straw in a concentrate-rich diet: insights into microbe-host interactions
Source: mSystems. 2024 Sep 17;9(10):e00483-24. doi: 10.1128/msystems.00483-24 (PMC11494937; doi:10.1128/msystems.00483-24)
Supplement: Supplemental Figures — Figures S1 to S4. [file msystems.00483-24-s0001.pdf]

Differential immunological responses in lamb rumen and colon to alfalfa hay and wheat straw in a concentrate-rich diet: Insights into microbe-host interactions

Kefyalew Gebeyew[1,2], Hui Mi [1,2], Yong Liu[1,2], Yongbin Liu[3*], Biao Wang[4], Teka Feyera[5], Zhiliang Tan[1,2], Zhixiong He[1,2*]

[1]CAS Key Laboratory for Agro-Ecological Processes in Subtropical Region, National Engineering Laboratory for Pollution Control and Waste Utilization in Livestock and Poultry Production, Hunan Provincial Key Laboratory of Animal Nutritional Physiology and Metabolic Process, Institute of Subtropical Agriculture, Chinese Academy of Sciences, Changsha, Hunan 410125, China

[2]University of Chinese Academy of Sciences, Beijing 100049, China

[3]School of Life Sciences, Inner Mongolia University, Hohhot, Inner Mongolia 010031, China

[4]Inner Mongolia Academy of Agricultural and Animal Husbandry Sciences, Hohhot, Inner Mongolia 010031, China

[5]Animal Science, School of Environmental and Rural Science, University of New England, Armidale, NSW 2351, Australia

* Address correspondence to: Yongbin Liu; Zhixiong He

Email: ybliu@imu.edu.cn; zxhe@isa.ac.cn;

Supplementary figure

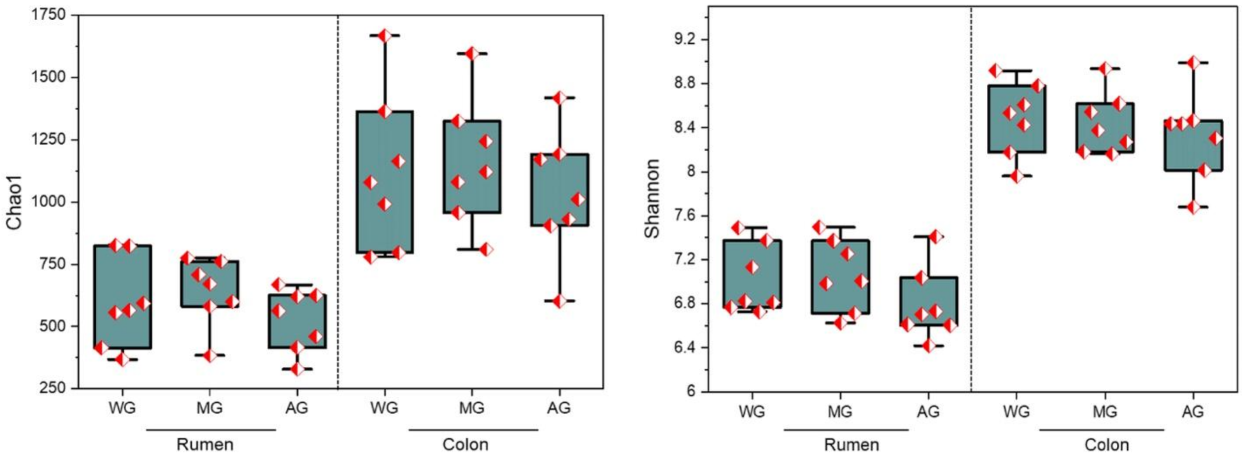

Figure S1. Effects of alfalfa hay, wheat straw, and their mixture supplemented with a high-grain-based diet on the alpha diversity (Chao1 and Shannon Indexes) of microbiota from the rumen and colon contents.

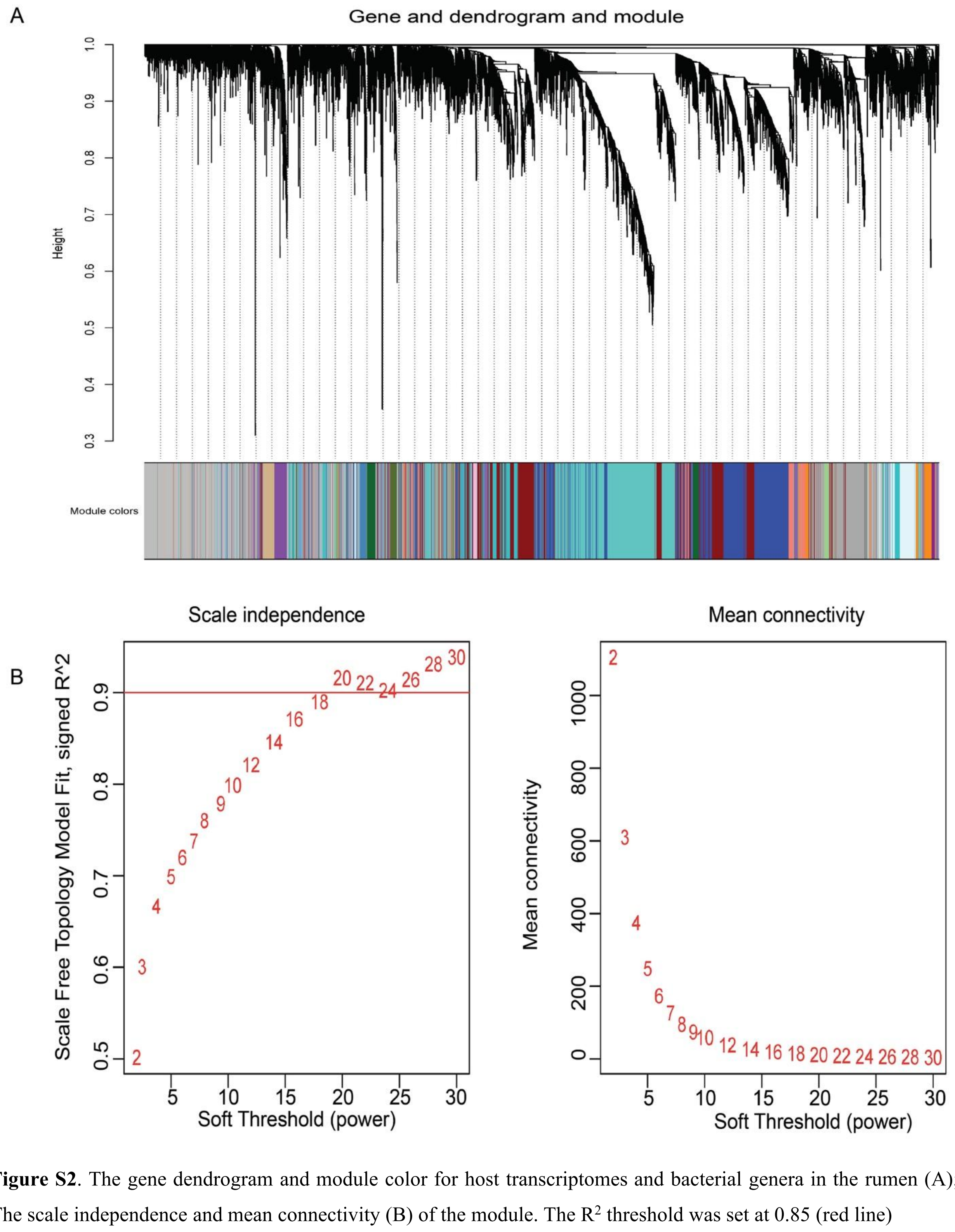

Figure S2. The gene dendrogram and module color for host transcriptomes and bacterial genera in the rumen (A); The scale independence and mean connectivity (B) of the module. The R^2 threshold was set at 0.85 (red line)

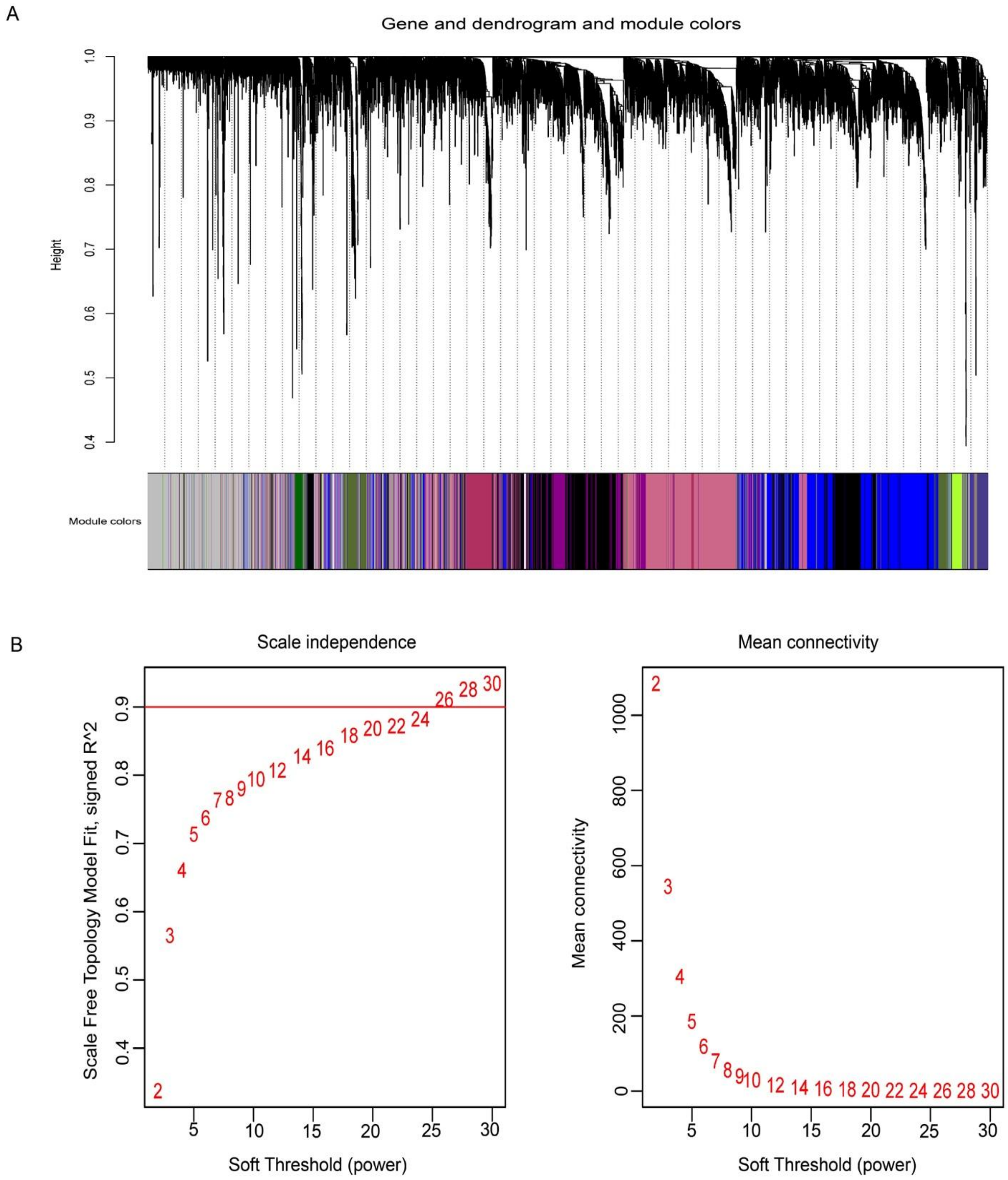

Figure S3. The gene dendrogram and module color for host transcriptomes and bacterial genera in the colon (A); The scale independence and mean connectivity (B) of the module. The R^2 threshold was set at 0.85 (red line)

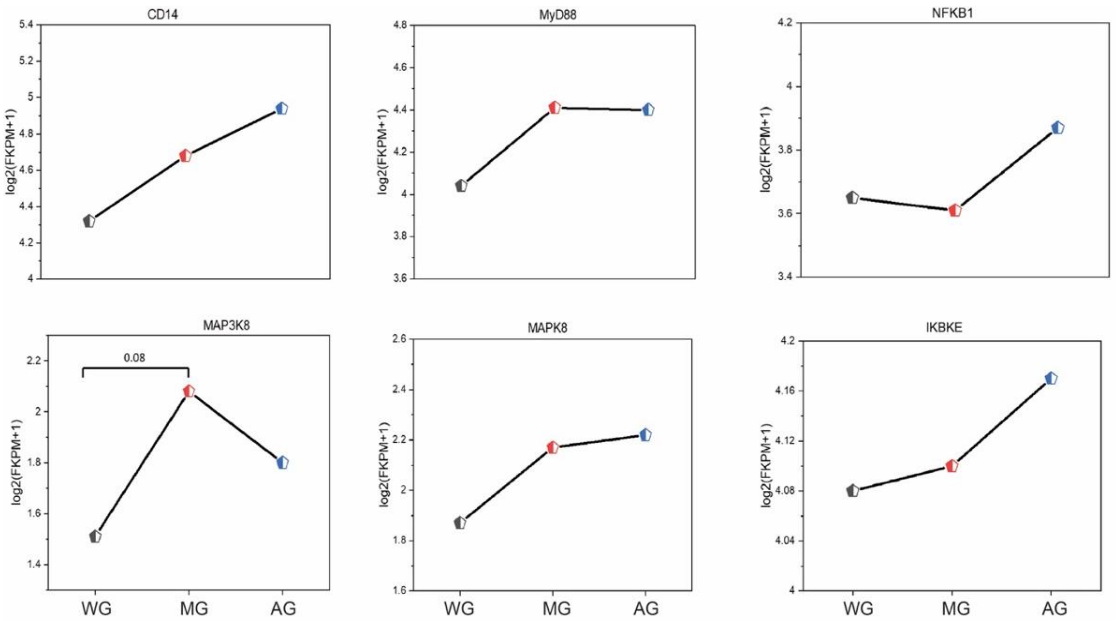

Fig. 4. The expression ($\log_2$(FKPM +1)) of genes showed negative or positive associations with the individual bacterial genera in the rumen. Genes involved in significantly enriched pathways in the light-cyan module and had a significant association with the individual bacterial genera ($P < 0.05$) were displayed.
