# Supplementary material for: Differential immunological responses in lamb rumen and colon to alfalfa hay and wheat straw in a concentrate-rich diet: insights into microbe-host interactions
Source: mSystems. 2024 Sep 17;9(10):e00483-24. doi: 10.1128/msystems.00483-24 (PMC11494937; doi:10.1128/msystems.00483-24)
Supplement: Graphical Abstract — How alfalfa hay and wheat straw alter the colon bacteria and epithelium in lambs. [file msystems.00483-24-s0002.pdf]

Graphical Abstract

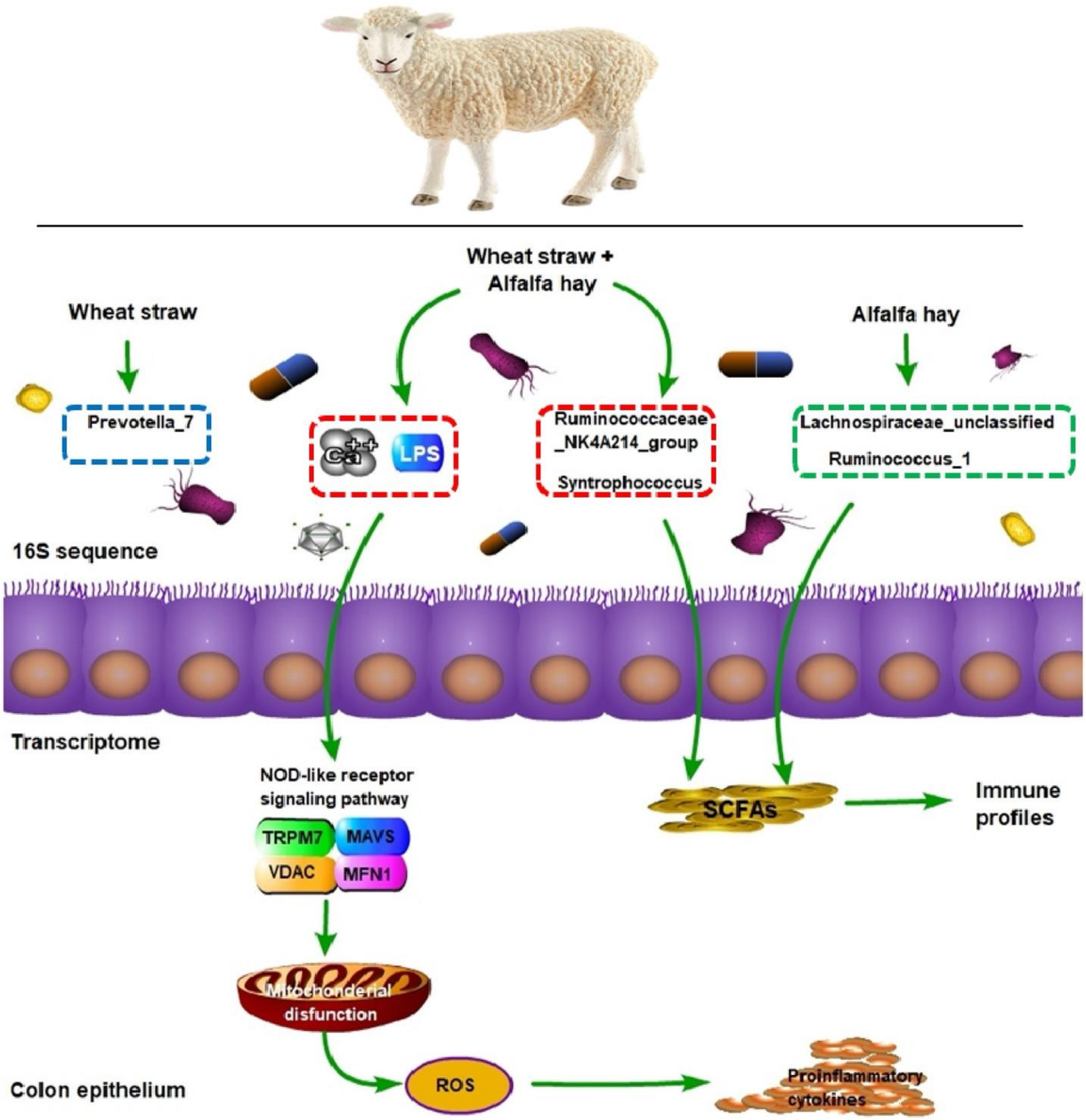

Systematic illustration of how alfalfa hay and wheat straw alone or combined alters the colon bacteria and epithelium in lambs fed in a HGB diet.
